# Supplementary material for: The causal relationship between the human gut microbiota and pyogenic arthritis: a Mendelian randomization study
Source: Front Cell Infect Microbiol. 2024 Nov 26;14:1452480. doi: 10.3389/fcimb.2024.1452480 (PMC11629706; doi:10.3389/fcimb.2024.1452480)
Supplement: Supplementary file 1 [file DataSheet1.zip › mendelian test/ebi-a-GCST90027728.csv_forest.pdf]

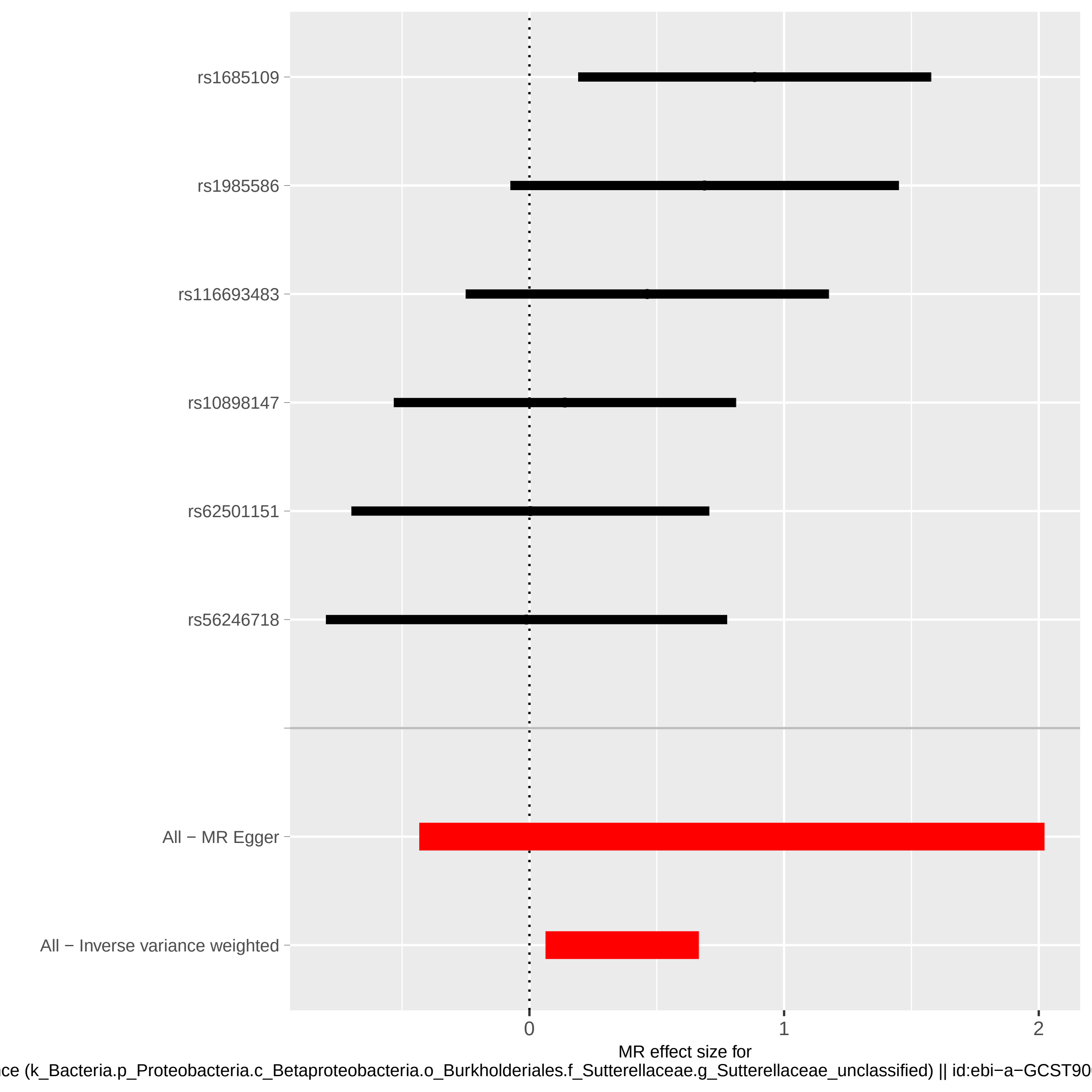

rs1685109
rs1985586
rs116693483
rs10898147
rs62501151
rs56246718
All − MR Egger
All − Inverse variance weighted
0
1
2
MR effect size for
nce (k_Bacteria.p_Proteobacteria.c_Betaproteobacteria.o_Burkholderiales.f_Sutterellaceae.g_Sutterellaceae_unclassified) || id:ebi−a−GCST90
